# Glucose metabolism status modifies the relationship between lipoprotein(a) and carotid plaques in individuals with fatty liver disease

**DOI:** 10.3389/fendo.2022.947914

**Published:** 2022-11-16

**Authors:** Jiaxuan Wang, Honglin Sun, Ying Wang, Yu An, Jia Liu, Guang Wang

**Affiliations:** ^1^ Department of Endocrinology, Beijing Chao-Yang Hospital, Capital Medical University, Beijing, China; ^2^ Physical Examination Center, Beijing Chao-Yang Hospital, Capital Medical University, Beijing, China

**Keywords:** lipoprotein(a), impaired fasting glucose, diabetes, carotid plaques, fatty liver disease

## Abstract

**Background and aims:**

Glucose and lipoprotein(a) [Lp(a)] have been recognized risk factors for atherosclerosis. The impact of both factors on fatty liver patients has not been studied. The aim of this study is to explore the role of high-level Lp(a) and different glucose metabolism statuses on carotid plaques in fatty liver patients.

**Methods:**

We selected 4,335 fatty liver patients in this cross-sectional study. The diagnosis of fatty liver disease and carotid plaques was made by ultrasound. Participants were divided into four groups based on glucose metabolism status (normal glucose regulation [NGR], lower bound of impaired fasting glucose [IFG-L], higher bound of impaired fasting glucose [IFG-H], diabetes mellitus [DM]) and then categorized into 12 subgroups according to Lp(a) concentrations. The association between variables was estimated by odds ratio (OR).

**Results:**

Carotid plaques were present in 1,613 (37.2%) fatty liver patients. Lp(a)≥30 mg/dL was associated with high risk of carotid plaques in those patients with IFG-L, IFG-H and DM (OR 1.934 [95% CI 1.033-3.618], 2.667 [1.378-5.162], 4.000 [2.219-7.210], respectively; *p*<0.05). Fatty liver patients with DM plus Lp(a)<10 mg/dL and 10≤Lp(a)<30 mg/dL were more vulnerable to carotid plaques (OR 1.563 [95% CI 1.090-2.241], 1.930 [1.279-2.914]), respectively, *p*<0.05).

**Conclusions:**

Our study first suggested that high-level Lp(a) may raise the risk of carotid plaques in fatty liver patients with not only diabetes but also IFG, manifesting that Lp(a) may be helpful for the early discovery of subclinical atherosclerosis in fatty liver patients with impaired glucose metabolism.

## Introduction

The disease with the highest morbidity and mortality is still cardiovascular disease (CVD) (1). The subclinical vascular disease represents a variety of pathological vascular changes that occur before the clinical signs of CVD, providing momentous etiological insights into the early detection of CVD progression (2). The atherosclerotic plaque is a great indicator of subclinical atherosclerosis by the ultrasound, further assessing cardiovascular risk (3).

When the ectopic fat accumulates (≥5%) in liver cells, it is considered the fatty liver disease (FLD), which becomes the major contributor to chronic liver disease (4). There is a growing prevalence of 2%-44% in ordinary people (5). Its pathological classification included simple steatosis, steatohepatitis, cirrhosis and even hepatocellular carcinoma (6). Several studies have indicated that FLD had a close relationship with CVD and subclinical atherosclerosis (7, 8).

The primary public health crisis includes type 2 diabetes, and China has the world’s largest diabetic population (9). The principal causes of morbidity and mortality among patients with type 2 diabetes are cardiovascular complications (10). Moreover, the great prevalence of pre-diabetes is expected to transform into an enormous burden of diabetes and relevant CVD in the future (9).

Lipoprotein(a) [Lp(a)] is produced in the liver. It is characterized in that the apolipoprotein B100 molecule is covalently bound to the glycoprotein apolipoprotein (a) (11). Lp(a) belongs to a subgroup of low-density lipoproteins, and the LPA gene genetically determines the concentration of Lp(a) (12). It plays important roles in the development of atherosclerosis and thrombosis as it is similar to low-density lipoprotein cholesterol (LDL-C) and plasminogen (13). A higher level of Lp(a) is linked to an increased risk of coronary, peripheral artery, cerebrovascular disease events and carotid atherosclerosis (14–19). Besides, a negative association of lipoprotein(a) with the prevalence of diabetes have been found in populations with or without the evident cardiovascular disease (20–24). Nevertheless, the intervening node of increased Lp(a) for individuals with abnormal glucose metabolism and CVD has not been determined yet in an authoritative guide.

Few studies to date have unveiled the impact of Lp(a) on the risk of carotid plaques in FLD with glucose metabolism disorders. In our cross-sectional study, we aimed to explore the relationship between lipoprotein(a) and carotid plaques in fatty liver disease individuals with different glucose metabolism.

## Materials and methods

### Study population

**Figure 1:**
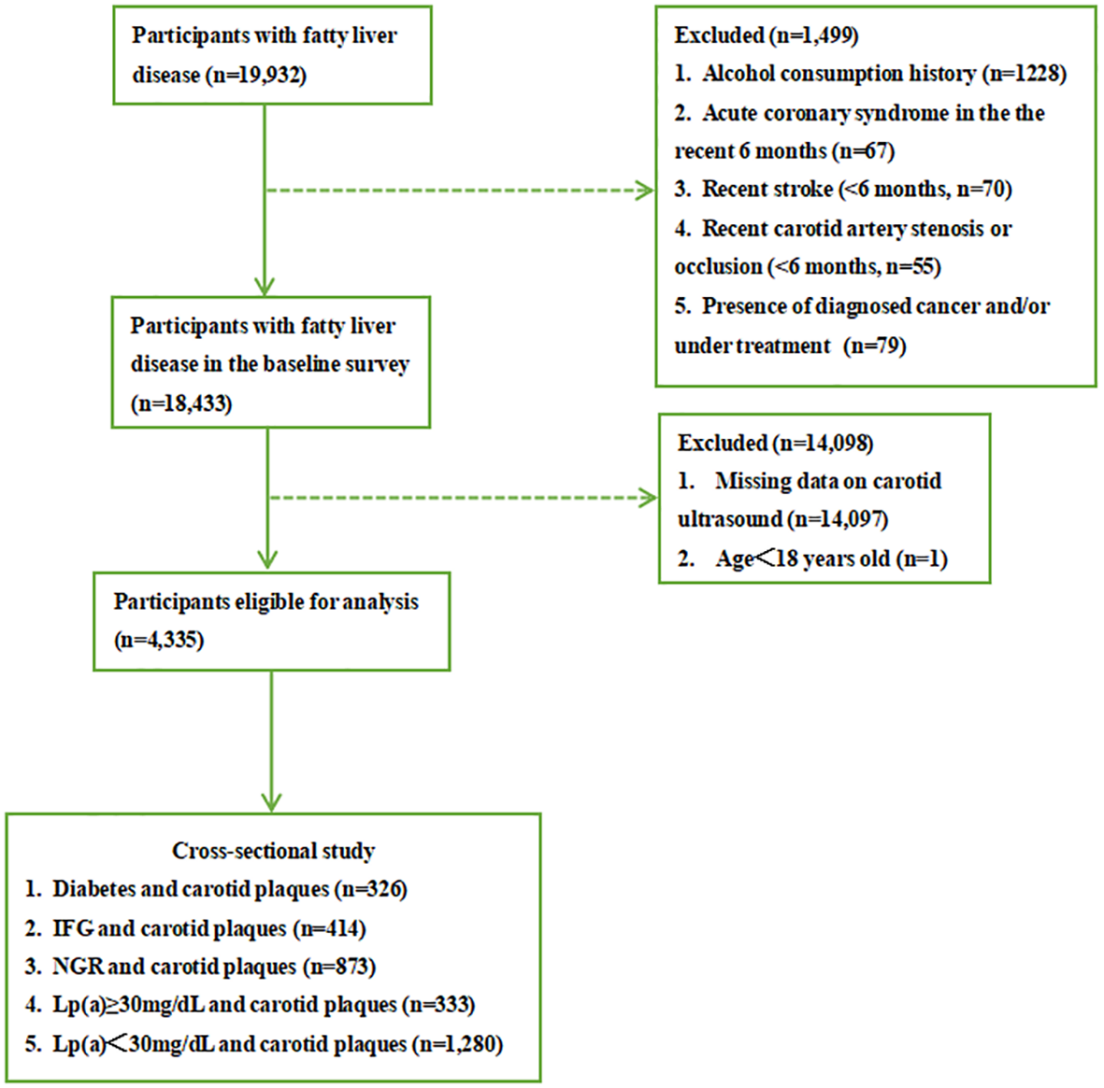
Flowchart of study procedure. IFG, impaired fasting glucose; NGR, normal glucose regulation.

**Figure 1** shows the flowchart of the research procedure. A total of 19,932 patients with fatty liver were diagnosed by abdominal ultrasonography in the physical examination center of Beijing Chao-Yang Hospital of China from April 2016 to August 2021. We excluded participants with alcohol consumption history (≥20 g a day for women and ≥30 g a day for men (6), n=1228), acute coronary syndrome in the recent 6 months (n=67), recent stroke (n=70), recent carotid artery stenosis or occlusion (<6 months, n=55), and presence of diagnosed cancer and/or under treatment (n=79). Then 1,4097 patients who did not perform carotid ultrasonography and one whose age was under 18 years old were excluded as well. At last, 4,335 patients were included in the study who concurrently measured serum Lp(a). All participants signed written informed consent. The research protocol complies with the ethical guidelines of the 1975 Declaration of Helsinki. It was approved by the Ethics Committee of Beijing Chao-Yang Hospital.

### Measurements

The experienced technicians measured the body height, weight, systolic blood pressure (SBP), and diastolic blood pressure (DBP) of the participants according to a standard protocol. Body mass index (BMI) was the ratio of weight in kilograms to the square of height in meters (kg/m^2^). All blood tests were determined by standard laboratory procedures, including Lp(a), fasting blood glucose (FBG), fasting insulin (FINS), glycosylated hemoglobin (HbA1c), glycated albumin (GA), total cholesterol (TC), LDL-C, high-density lipoprotein cholesterol (HDL-C), triglycerides (TG), total protein (TP), albumin (ALB), alanine aminotransferase (ALT), and aspartate aminotransferase (AST), gamma-glutamyl transpeptidase (GGT), total bilirubin (TBIL), direct bilirubin (DBIL), indirect bilirubin (IBIL), total bile acid (TBA), creatinine (Cr), blood urea nitrogen (BUN), uric acid (UA) and free fatty acid (FFA).

Patients have regular physical exams at our health management center, where carotid and abdominal ultrasounds are performed. They were examined according to standardized protocols by trained sonographers. We used high-resolution B-mode for carotid and abdominal respectively, as described previously (25–28). Carotid plaques were defined as focal intima-media thickness ≥1.2 mm (29). Two experienced sonographers who were unacquainted with the participants’ states of illness measured the intima-media thickness. The diagnosis of FLD involved the diffuse hyperechogenicity in the liver in comparison to the kidney, vascular blurring, and deep attenuation of ultrasound signals (30).

### Definitions

The definitions of hypertension, DM, dyslipidemia, low bound of IFG (IFG-L), high bound of IFG (IFG-H), and normal glucose regulation (NGR) were as followings:

- Hypertension: SBP≥140 mmHg, or DBP≥90 mmHg (31), or previous diagnosis.
- Diabetes: FBG≥7 mmol/l, or HbA1C≥6.5% (32), or previous diagnosis, or current use of anti-diabetic drugs.
- Dyslipidemia: TC≥6.22 mmol/L, or TG≥2.26 mmol/L, or LDL-C≥4.14 mmol/L, or HDL-C<1.04 mmol/L (33), or previous diagnosis.
- IFG-L: FBG 5.6–6.0 mmol/L (34, 35).
- IFG-H: FBG 6.1–6.9 mmol/L (34, 35).
- NGR: FBG<5.6 mmol/L (34, 35).

### Statistics analysis

For continuous variables, the values were reported as the mean standard deviation or median (25th-75th percentile), and for categorical variables, the number (percentage). To assess the distribution of the variables, the Kolmogorov-Smirnov test was utilized. The Student t-test, Mann-Whitney U test, or chi-square test were used to analyze the differences in clinical indicators between groups where appropriate. The prevalence of carotid plaques in the comparison among groups was further estimated by the Bonferroni test. Binary logistic regression analyses were performed to calculate the odds ratio (OR). The statistical significance was defined as a value of *p*<0.05. The SPSS software was used to conduct the statistical analysis (version 24.0).

## Results

### Baseline characteristics

**Table 1** shows the characteristics of patients with fatty liver. The age, SBP, DBP, glucose, HbA1c, GA, Lp(a), TBA, and BUN levels were linked to the presence of carotid plaques, while TP, ALB, and ALT had a negative correlation (*p*<0.001). The proportion of male participants and those with dyslipidemia was elevated when they suffered carotid plaques (*p*<0.001). But there was no discernible difference in BMI, FINS, TC, TG, HDL-C, LDL-C, FFA, AST, GGT, bilirubin, and UA between the two groups (*p*>0.05).

**Table 1:** Baseline characteristic of fatty liver patients with or without carotid plaques.

Variable	Without carotid plaques	With carotid plaques	*P* value
M, %	1967 (72.3)	1290 (80.0)	<0.001
Age, years	46.9 ± 9.7	57.7 ± 9.5	<0.001
BMI, kg/m^2^	27.54 ± 3.31	27.39 ± 3.02	0.129
SBP, mmHg	128 ± 16	136 ± 18	<0.001
DBP, mmHg	78 ± 12	80 ± 12	<0.001
FBG, mmol/L	5.11 (4.71, 5.67)	5.49 (5.00, 6.58)	<0.001
HbA1c, %	5.6 (5.4, 5.9)	5.9 (5.6, 6.5)	<0.001
FINS, mIU/L	12.15 (8.70,16.68)	11.80 (8.43, 16.20)	0.398
GA, %	13.0 (12.1, 14.2)	13.7 (12.6, 15.6)	<0.001
TC, mmol/L	5.09 ± 0.94	5.13 ± 1.08	0.223
HDL-C, mmol/L	1.15 ± 0.25	1.16 ± 0.26	0.507
LDL-C, mmol/L	3.1 (2.6, 3.7)	3.2 (2.5, 3.8)	0.197
TG, mmol/L	1.80 (1.31, 2.58)	1.83 (1.35, 2.53)	0.321
Lp (a), mg/dL	10.9 (6.4, 20.8)	12.4 (7.2, 24.8)	<0.001
TP, g/L	75.2 (72.6, 77.9)	74.4 (71.8, 77.1)	<0.001
ALB, g/L	46.67 ± 2.48	46.10 ± 2.37	<0.001
AST, U/L	23.0 (20.0, 28.0)	23.0 (19.5, 28.0)	0.360
ALT, U/L	27 (20, 39)	25 (19, 35)	<0.001
GGT, U/L	32 (22, 50)	33 (22, 52)	0.597
TBIL, umol/L	13.9 (10.9, 17.8)	14.1 (11.1, 17.6)	0.274
DBIL, umol/L	4.20 (3.20, 5.37)	4.20 (3.30, 5.40)	0.590
IBIL, umol/L	9.60 (7.46, 12.60)	9.86 (7.70, 12.50)	0.212
TBA, umol/L	3.4 (2.3, 5.1)	3.8 (2.5, 5.7)	<0.001
Cr, umol/L	69.05 (58.50, 77.80)	69.2 (60.7, 78.2)	0.007
BUN, mg/dL	5.18 (4.45, 6.03)	5.51 (4.75, 6.35)	<0.001
UA, umol/L	388 (330, 448)	385 (328, 442)	0.261
FFA, mmol/L	0.54 (0.41, 0.66)	0.55 (0.43, 0.68)	0.076
Hypertension, %	667 (25.6)	629 (40.8)	<0.001
Diabetes, %	328 (15.2)	444 (32.7)	<0.001
Dyslipidemia, %	1614 (59.3)	1038 (64.4)	0.001

Data are expressed as mean ± standard deviation, median (25th-75th percentile) or the number (percentage). M, male; BMI, body mass index; SBP, systolic blood pressure; DBP, diastolic blood pressure; Lp (a), lipoprotein (a); FBG, fasting blood glucose; FINS, fasting insulin; HbA1c, glycosylated hemoglobin; GA, glycated albumin; TC, total cholesterol; LDL-C, low-density lipoprotein cholesterol; HDL-C, high-density lipoprotein cholesterol; TG, triglycerides; TP, total protein; ALB, albumin; ALT, alanine aminotransferase; AST, aspartate aminotransferase; GGT, gamma-glutamyl transpeptidase; TBIL, total bilirubin; DBIL, direct bilirubin; IBIL, indirect bilirubin; TBA, total bile acid; Cr, creatinine; BUN, blood urea nitrogen; UA, uric acid; FFA, free fatty acid.

### Glucose metabolism, Lp(a) levels, and carotid plaques in fatty liver patients

**Figure 2:**
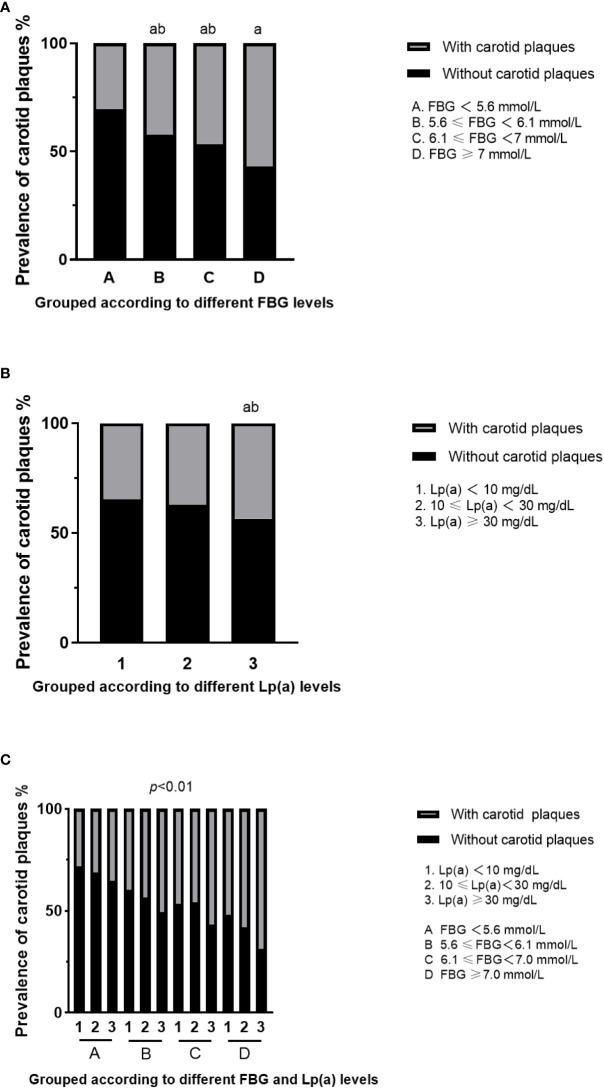
Prevalence of carotid plaques in fatty liver patients with different FBG and Lp(a) levels. **(A)** Prevalence of carotid plaques in fatty liver patients grouped according to different FBG levels. ^a^For *p*<0.05 vs A group (FBG<5.6 mmol/L), ^b^for *p*<0.05 vs D group (FBG>7.0 mmol/L). **(B)** Prevalence of carotid plaques in fatty liver patients grouped according to different Lp(a) levels. ^a^For *p*<0.05 vs 1 group (Lp(a)<10 mg/dL), ^b^for *p*<0.05 vs 2 group (10≤Lp(a)<30 mg/dL). **(C)** Prevalence of carotid plaques in fatty liver patients with both different FBG and Lp(a) levels. FBG, fasting blood glucose; Lp(a), lipoprotein(a).

The prevalence of carotid plaques in the four groups divided by different fasting blood glucose levels (NGR, IFG-L, IFG-H, and DM) was 30.7%, 43.1%, 48.1%, and 56.9%, respectively (**Figure 2A**). According to the Pearson chi-square test, the presence of carotid plaques in fatty liver patients rose with FBG levels (*p*<0.05). Individuals with Lp(a)≥30 mg/dL were at the greatest risk of carotid plaques among three groups divided by Lp(a) level (Lp(a)<10 mg/dL, 10≤Lp(a)<30 mg/dL, and Lp(a)≥30 mg/dL) (**Figure 2B**). However, when the patients were assessed in line with glycometabolism and Lp(a) level, the individuals with fatty liver and Lp(a)≥30 mg/dL, regardless of different glucose metabolism statuses, had a strikingly increased risk of carotid plaques compared to the reference subjects (NGR and Lp(a)<10 mg/dL). Meanwhile, the prevalence risk of carotid plaques in the groups of DM plus 10≤Lp(a)<30 mg/dL, DM plus Lp(a)≥30 mg/dL, and IFG-H plus Lp(a)≥30 mg/dL became higher than the reference subjects (**Figure 2C**) (*p*<0.05).

The binary logistic regression crude models in **Table 2** indicated that fatty liver individuals with DM plus Lp(a)≥30 mg/dL were at a 5.810-fold increased risk of carotid plaques compared to reference subjects (OR 5.810 [95% CI 3.726-9.059], *p*<0.05). After confounding variables were adjusted, like age, sex, ALB, ALT, TBA, creatinine, hypertension, and dyslipidemia, the association between high-level Lp(a) and carotid plaques in participants with DM remained significant. The presence risk of carotid plaques in those individuals with FBG<7mmol/L plus Lp(a)<30 mg/dL did not increase in comparison to the reference individuals (*p*>0.05). Besides, in the group of NGR, the fatty liver people with Lp(a)≥30 mg/dL were not inclined to suffer carotid plaques. In addition, for each standard deviation increase in Lp(a) level in the DM group (25.7 mg/dL), the risk for carotid plaques went up by 35.4%. Patients with DM plus Lp(a)<10 mg/dL and DM plus 10≤Lp(a)<30 mg/dL were at 1.563-fold (95% CI 1.090-2.241) and 1.930-fold (95% CI 1.279-2.914) high risk of carotid plaques (*p*<0.05). Individuals with high-level Lp(a) had a relationship with 1.934, 2.667, and 4.000-fold risk of carotid plaques in the three groups with different glucose metabolism (IFG-L, IFG-H, and DM), respectively.

**Table 2:** Lp(a) levels in relation to carotid plaques in fatty liver patients with different glucose metabolism.

Lp(a)(mg/dL)	With carotid plaques/subjects(1613/4335)	Crude model OR(95%CI)	Adjusted model OR(95%CI)
**FBG<5.6mmol/L**			
Lp(a) per SD increase		1.124 (1.039, 1.215)^a^	1.083 (0.971, 1.207)
Lp(a)<10	335/1197	Ref	Ref
10≤Lp(a)<30	359/1144	1.210 (1.008, 1.452)^a^	1.074 (0.836, 1.380)
Lp(a)≥30	179/506	1.427 (1.135, 1.795)^a^	1.341 (0.976, 1.842)
**5.6≤FBG<6.1mmol/L**			
Lp(a) per SD increase		1.118 (0.927, 1.348)	1.070 (0.846, 1.355)
Lp(a)<10	90/226	1.683 (1.226, 2.312)^a^	1.154 (0.742, 1.794)
10≤Lp(a)<30	90/207	2.012 (1.461, 2.771)^a^	1.290 (0.835, 1.992)
Lp(a)≥30	44/87	2.577 (1.600, 4.152)^a^	1.934 (1.033, 3.618)^a^
**6.1≤FBG<7.0mmol/L**			
Lp(a) per SD increase		1.247 (1.454, 2.831)^a^	1.295 (0.997, 1.683)
Lp(a)<10	83/179	2.029 (1.527, 3.140)^a^	1.305 (0.834, 2.041)
10≤Lp(a)<30	65/142	2.189 (1.525, 3.093)^a^	1.011 (0.623, 1.642)
Lp(a)≥30	42/74	3.667 (2.178, 6.174)^a^	2.667 (1.378, 5.162)^a^
**FBG≥7.0mmol/L**			
Lp(a) per SD increase		1.251 (1.036, 1.512)^a^	1.354 (1.066, 1.719)^a^
Lp(a)<10	146/281	2.864 (2.192, 3.744)^a^	1.563 (1.090, 2.241)^a^
10≤Lp(a)<30	112/193	3.662 (2.675, 5.013)^a^	1.930 (1.279, 2.914)^a^
Lp(a)≥30	68/99	5.810 (3.726, 9.059)^a^	4.000 (2.219, 7.209)^a^

Model adjusted for age, sex, ALB, ALT, TBA, Cr, hypertension, and dyslipidemia. ^a^For p<0.05. ALB, albumin; ALT, alanine aminotransferase; TBA, total bile acid; Cr, creatinine.

## Discussion

In our research, we analyzed the impact of Lp(a) on the prevalence risk of carotid plaques in fatty liver patients with four groups of various glucose metabolism. Our main findings showed that the fatty liver population with diabetes plus Lp(a)≥30 mg/dL were at a quadruple higher risk of carotid plaques in comparison to the reference subjects. Besides, the carotid plaques risk in those with IFG plus Lp(a)≥30 mg/dL was higher than in the fatty liver participants with diabetes plus Lp(a)<30 mg/dL, indicating that measurement of Lp(a) was significant in the patients including not only diabetes but also the pre-diabetes.

Although the apparent vascular disease had not appeared in the patients with carotid atherosclerosis, they had more risk of CVD than the ones without carotid atherosclerosis (26, 36). Among individuals with non-alcoholic fatty liver disease (NAFLD), DM was connected with prevalent subclinical atherosclerosis evaluated by the detection of carotid plaques (37). That was in keeping with our present findings that fatty liver patients with rising FBG were more likely to suffer carotid plaques. There were similar risk factors in diabetes and NAFLD, and both diseases were closely related consequently. The simultaneous presence of both NAFLD and type 2 diabetes exacerbated lipid disorders as well as hepatic insulin resistance, in turn worsening atherosclerosis and raising the incidence of cardiovascular events among type 2 diabetes individuals (38–40).

Substantial evidence suggested that the determining factor of residual CVD risk might be Lp(a) when LDL-C level was optimum (41). Even though target LDL-C levels were reached, the increasing concentration of Lp(a) remained relevant to the existence of carotid atherosclerosis (19). The recent studies indicated that higher Lp(a) level was associated with subclinical coronary atherosclerosis in asymptomatic subjects (42), and elevated baseline Lp(a) was associated with subclinical vascular and valvular calcification in the White and Black participants (43). It is known that Lp(a) and its associated oxidized phospholipids could induce a proinflammatory response, causing cellular apoptosis and necrosis which accelerate necrotic core formation (44, 45). Furthermore, Lp(a) contains proatherogenic components of LDL, and its prothrombotic effects through the plasminogen-like apolipoprotein(a) also contribute to the atherosclerosis (46, 47). Our results also manifested that high Lp(a) levels lead to an increased risk of subclinical atherosclerosis which was consistent with previous studies. As fatty liver disease, diabetes, and Lp(a) levels had a link with the presence of carotid plaques dissimilarly based on earlier reports, it is worthy of exploring the forecast value of Lp(a) in various subpopulations.

We studied the link between serum Lp(a) and carotid plaques in fatty liver patients with different glucose metabolism. We found that the risks for carotid plaques whose FBG≥7 mmol/L reached the highest among the whole study population when grouped by FBG levels (NGR, IFG-L, IFG-H, and DM) through binary logistic regression analysis. When grouped by different concentrations of FBG and Lp(a), patients with IFG-L plus Lp(a)≥30 mg/dL and IFG-H plus Lp(a)≥30 mg/dL were at 1.934 and 2.667-fold higher risk of carotid plaques than the reference groups respectively. In contrast, the risk prediction was unaffected with the addition of Lp(a) when FBG<5.6 mmol/L. It was the first time to indicate that fatty liver individuals suffering from IFG and high Lp(a) levels simultaneously were at an increased risk of carotid plaques. Pre-diabetes population with elevated Lp(a) levels were more vulnerable to cardiovascular events, and the diabetic population with high-level Lp(a) had the worst prognosis in different countries and races (48–50), which was similar to our results. However, several studies reported that plasma Lp(a) decreased in T2DM patients compared to controls, which is called Lp(a) paradox in T2DM (51). It is necessary to measure Lp(a) concentrations in diabetes patients and evaluate the risk considering the paradox effect. A study had found that lowering the Lp(a) levels from above 50 nmol/l to 14 nmol/l could reduce risk of CVD and would not increase risk of T2DM (20), but guidelines for measurements or treatments of high-Lp(a) levels in diabetes or prediabetes patients have not been published yet at present.

There were several limitations in this study including that only Chinese patients were chosen as candidates and whether the other races could show the same tendency needs further investigation. While ultrasound is simple, harmless and reproducible, we did not perform liver biopsy which is the gold standard for the diagnosis of fatty liver disease. We could not identify the patients with or without non-alcoholic steatohepatitis as well. In addition, the data on smoking and family history were not gathered, and more research is required with the data taken into account. The history of hepatitis had little influence on our study as the infection rate of Hepatitis B Virus and Hepatitis C Virus in China was only 6.89% from 2013 to 2017 (52). Beyond that, due to the cross-sectional design of our study, we did the research only at baseline and found the relationship between Lp(a), glucose metabolism and carotid plaques at one point of time. Further research is necessary to explore more robust evidence from prospective studies.

## Conclusions

Our cross-sectional study indicated that fatty liver patients with diabetes and high-level Lp(a) were the most vulnerable to carotid plaques. But more than that, we first suggested that the the presence of carotid plaques was closely related to the high-level Lp(a) and IFG in the fatty liver patients, implying that testing of Lp(a) and treatment towards high-level Lp(a) might make sense for fatty liver patients with not merely DM but mildly impaired glucose metabolism.

## Data availability statement

The raw data supporting the conclusions of this article will be made available by the authors, without undue reservation.

## Ethics statement

The studies involving human participants were reviewed and approved by Medical Ethics Committee, Beijing Chaoyang Hospital, Capital Medical University. The patients/participants provided their written informed consent to participate in this study.

## Author contributions

YW gathered the data of the participants. YA further organized the data. JXW and HLS conducted the design of the research, data analyses, and statistical analyses. JXW wrote the article, and prepared the presentation parts, all supervised by JL and GW. All authors contributed to the article and approved the submitted version.

## Conflict of interest

The authors declare that the research was conducted in the absence of any commercial or financial relationships that could be construed as a potential conflict of interest.

## Publisher’s note

All claims expressed in this article are solely those of the authors and do not necessarily represent those of their affiliated organizations, or those of the publisher, the editors and the reviewers. Any product that may be evaluated in this article, or claim that may be made by its manufacturer, is not guaranteed or endorsed by the publisher.

## Abbreviations

CVD, cardiovascular disease; DM ,diabetes; Lp(a), Lipoprotein(a); IFG, impaired fasting glucose; FLD, fatty liver disease; NAFLD, non-alcoholic fatty liver disease; LDL-C, low-density lipoprotein cholesterol; SBP, systolic blood pressure; DBP, diastolic blood pressure; BMI, body mass index; FBG, fasting blood glucose; FINS, fasting insulin; HbA1c, glycosylated hemoglobin; GA, glycated albumin; TC, total cholesterol; HDL-C, high-density lipoprotein cholesterol; TG, triglycerides; TP, total protein; ALB, albumin; ALT, alanine aminotransferase; AST, aspartate aminotransferase; GGT, gamma-glutamyl transpeptidase; TBIL, total bilirubin; DBIL, direct bilirubin; IBIL, indirect bilirubin; TBA, total bile acid; Cr, creatinine; BUN, blood urea nitrogen; UA, uric acid; FFA, free fatty acid; IFG-L, lower bound of impaired fasting glucose; IFG-H, higher bound of impaired fasting glucose; NGR, normal glucose regulation; OR, odds ratio.
